# Damped propagation of cell polarization explains distinct PCP phenotypes of epithelial patterning

**DOI:** 10.1038/srep02528

**Published:** 2013-09-02

**Authors:** Hao Zhu, Markus R. Owen

**Affiliations:** 1 Bioinformatics Section, School of Basic Medical Sciences, Southern Medical University, Guangzhou, 510515, China; 2 Center for Mathematical Biology and Medicine, School of Mathematical Sciences, The University of Nottingham, Nottingham, NG7 2RD, UK

## Abstract

During epithelial patterning in metazoans cells are polarized in the plane of a tissue, a process referred to as planar cell polarity (PCP). Interactions between a few molecules produce distinct phenotypes in diverse tissues in animals from flies to humans and make PCP tightly associated with tissue and organ growth control. An interesting question is whether these phenotypes share common traits. Previous computational models revealed how PCP signalling determines cell polarization in some specific contexts. We have developed a computational model, examined PCP signalling in varied molecular contexts, and revealed how details of molecular interactions and differences in molecular contexts affect the direction, speed, and propagation of cell polarization. The main finding is that damped propagation of cell polarization can generate rich variances in phenotypes of domineering non-autonomy and error correction in different contexts. These results impressively demonstrate how simple molecular interactions cause distinct, yet inherently analogous, developmental patterning.

Epithelial cells in many tissues are polarized not only along the apical-basal axis but also along the plane of tissues. This process, referred to as planar cell polarity (PCP), is essential for both tissue growth and patterning (reviewed in^1^). In *Drosophila*, a variety of PCP phenotypes have been observed, including the orientation of ommatidia in the eye and the direction of hairs and bristles in the wing and abdomen. In other metazoans, especially in insects, intriguing PCP phenotypes were reported decades ago^2^,^3^. Dynamic spatial cellular patterns within proliferating tissues also occur in plants^4^. Components in the PCP pathway are evolutionarily conserved (reviewed in^5^,^6^) and show a three-tiered hierarchy^7^. With Fat (Ft), Dachsous (Ds), and Four-jointed (Fj) as key components, those in the top layer produce a directional cue for PCP and control both cell polarization and tissue growth^8^,^9^,^10^. Those in the middle layer include three transmembrane proteins Frizzled (Fz)^11^, Strabismus (Stbm)^12^,^13^, and Flamingo (Fmi)^14^, and three cytoplasmic components Dishevelled (Dsh)^15^,^16^, Prickle (Pk)^17^,^18^, and Diego (Dgo)^19^. They receive, amplify, and propagate PCP signals. Those in the bottom layer control tissue-specific cellular patterning, such as hair differentiation in the skin and ommatidia rotation in the eye.

In *Drosophila*, during the first stage of PCP Fmi, Fz, and Dsh become uniformly localised around the cell perimeter^14^, and during the second stage these proteins adopt a proximodistal distribution within each cell (reviewed in^1^,^5^). Several feedback processes implemented by interactions between the above six components in the middle layer drive Fz and Dsh to move to the distal side and Stbm and Pk to the proximal side in each cell^18^ (Fig. 1). Fmi, a proto-cadherin important for interactions between Fz, Dsh, Pk, and Stbm, adopts a bipolar proximodistal distribution^14^. In *Drosophila's* wings and abdomen and vertebrates' skin, mutations in some top layer components (*ft* and *ds*) cause whorls and tufts of hairs (swirling patterns^20^,^21^), whereas mutations in some middle layer components (*fz* and *stbm*) change cell polarity not only in the mutant clone but also in some nearby wild-type cells (domineering non-autonomy^11^). Interactions between these components enable cell polarization to overcome small, but not big, errors in the directional cue^21^.

Experimental studies have revealed the basics about PCP (reviewed in^5^). Triggered by a directional cue, within each cell an initially slightly polarized Fz distribution or Fz activity drives Fz and Dsh to move toward the region of high Fz and Dsh concentrations via the intracellular Fz/Dsh interaction. Between cells the gradually polarized Fz and Stbm distributions drive Fz and Stbm to move toward each other via the intercellular Fz/Stbm interaction. These interactions form at least two positive feedbacks^5^,^18^. How the cue is determined in different tissues remains opaque (reviewed in^10^). Activities of Ft/Ds/Fj may determine the cue in some tissues^22^,^23^,^24^, but increasing evidence shows that Ft/Ds/Fj also guide cell polarization independently^25^,^26^. After initiation, interactions between, and movement of, molecules are driven by dynamic gradients of molecules (*driving forces*) and molecules' mobility in response to these gradients (*mobility*). These components finally obtain stable and polarized distributions indicated by hair directions in the skin and ommatidia orientations in the eye.

Mathematical models have been used to investigate different aspects of PCP (reviewed in^27^), including the Fz-initiated feedback amplifications^28^, the Fmi-mediated molecular distributions^29^, the intercellular Fz/Stbm interaction^30^, the impact of feedback and diffusion on PCP^31^, the mechanisms of swirling patterns^32^,^33^, the propagation of Fat signalling (in the Drosophila ovary)^34^, and to model PCP at different spatial scales^35^. Lack of biochemical data of PCP signalling made modellers estimate parameters (e.g., protein concentrations, binding affinities, reaction rates, and diffusion constants), yet these models re-produced experimentally observed PCP phenotypes in a robust way (insensitive to parameter changes).

Since these evolutionarily conserved components control many tissue-specific epithelial patterning, we wished to unveil whether distinct phenotypes share inherent commons. Our interest in this study was domineering non-autonomy and error correction. Two models addressed domineering non-autonomy^28^,^29^, but did not explore multiple directional cues^28^ (discussed several and examined two). The quantitative aspects of error correction have not been examined. Because disparate tissues in diverse animals should have varied directional cues, we explored domineering non-autonomy and error correction under different cues. Because details of biochemical reactions in different tissues remain too sparse for building detailed models, we developed a generic model that does not rely on such details as whether Fz and Stbm bind directly^28^ or via Fmi^29^ and whether molecules move by diffusion^28^,^29^ or via microtubules^36^,^37^. Because intercellular signalling was implemented via the Fz/Stbm interaction between cells, the homophilic cell adhesion molecule Fmi that biochemically facilitates and stabilises intercellular Fz/Stbm interaction^14^ was not explicitly included. Because Dgo binds to Dsh and promotes Fz signaling as the second distal cytoplasmic component^19^, it was also ignored. The concise model thus contains four key components - Fz, Dsh, Stbm, and Pk as representatives of distal transmembrane and cytoplasmic components and proximal transmembrane and cytoplasmic components. Similar to previous models^28^,^29^, an epithelial cell was divided into six compartments, the amount of each molecule was conserved in each cell during the simulated developmental period, and the model simulated cell polarization under cues during a limited time period without considering PCP's global reorientation^38^. This strategy - to use a concise model to explore fundamental properties of a signalling system - is supported by two arguments: 1) “the heart of the developmental signalling is not so much the individual molecules involved, but more the flow of information and the logic of the system they participate in”^39^, and 2) the behaviour of a full complex system must be constrained by the properties of its simpler core and is often well described by a simplified model^40^.

The implemented molecular interactions performed equally well and reproduced experimentally observed phenotypes under multiple directional cues. The results unveil considerable quantitative aspects of PCP, and some have not been adequately appreciated before. For example, changed speed of molecular movement may disproportionately affect the polarization of cells under weak and strong driving forces, which would cause aberrant cell polarization to propagate longer under a weaker directional cue and shorter under a strong one. We conclude that damped propagation (attenuated propagation with distance) of cell polarization is the common mechanism at the cell level to allow PCP signalling to produce distinct phenotypes of domineering non-autonomy and error correction.

## Results

### The model reproduced multiple experimentally observed phenotypes

**Figure 1:**
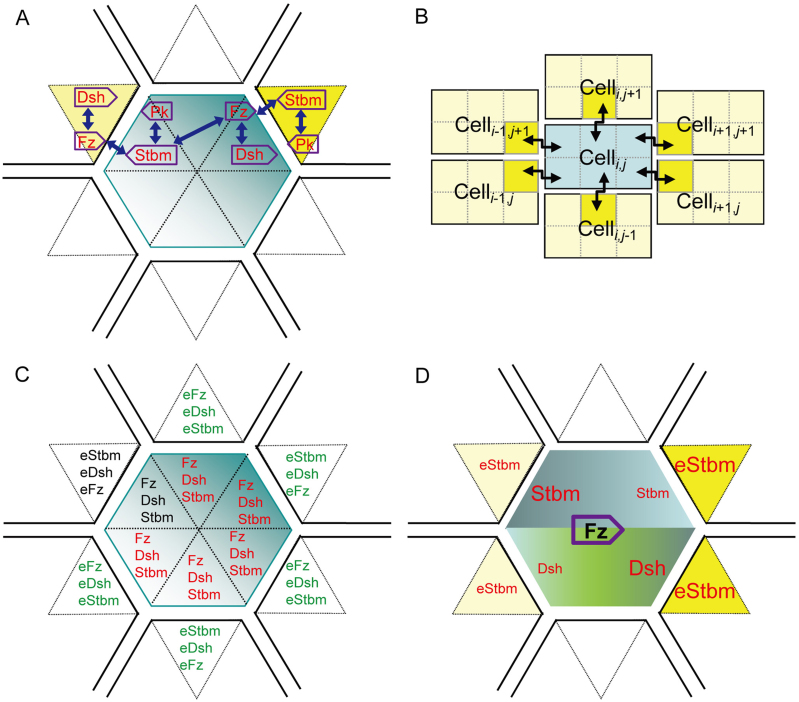
Schematic illustration of the model. Distal is to the right and proximal to the left in all figures. (A) Intra- and inter-cellular interactions (double arrows) between molecules drive the proximal and distal movement of Fz, Dsh, Stbm, and Pk (arrowed boxes) in each cell. (B) In a cell each compartment connects to a specific foreign compartment (marked in yellow). (C) Movement of a molecule (Fz, Dsh, and Stbm in black) is driven by the gradients of related molecules in the cell (Fz, Dsh, and Stbm in red) and in a specific foreign compartment (eFz, eDsh, and eStbm in black). (D) For example, Fz moves to compartments with high external Stbm and internal Dsh concentrations (indicated by big font size), and low internal Stbm concentration (indicated by small font size).

**Figure 2:**
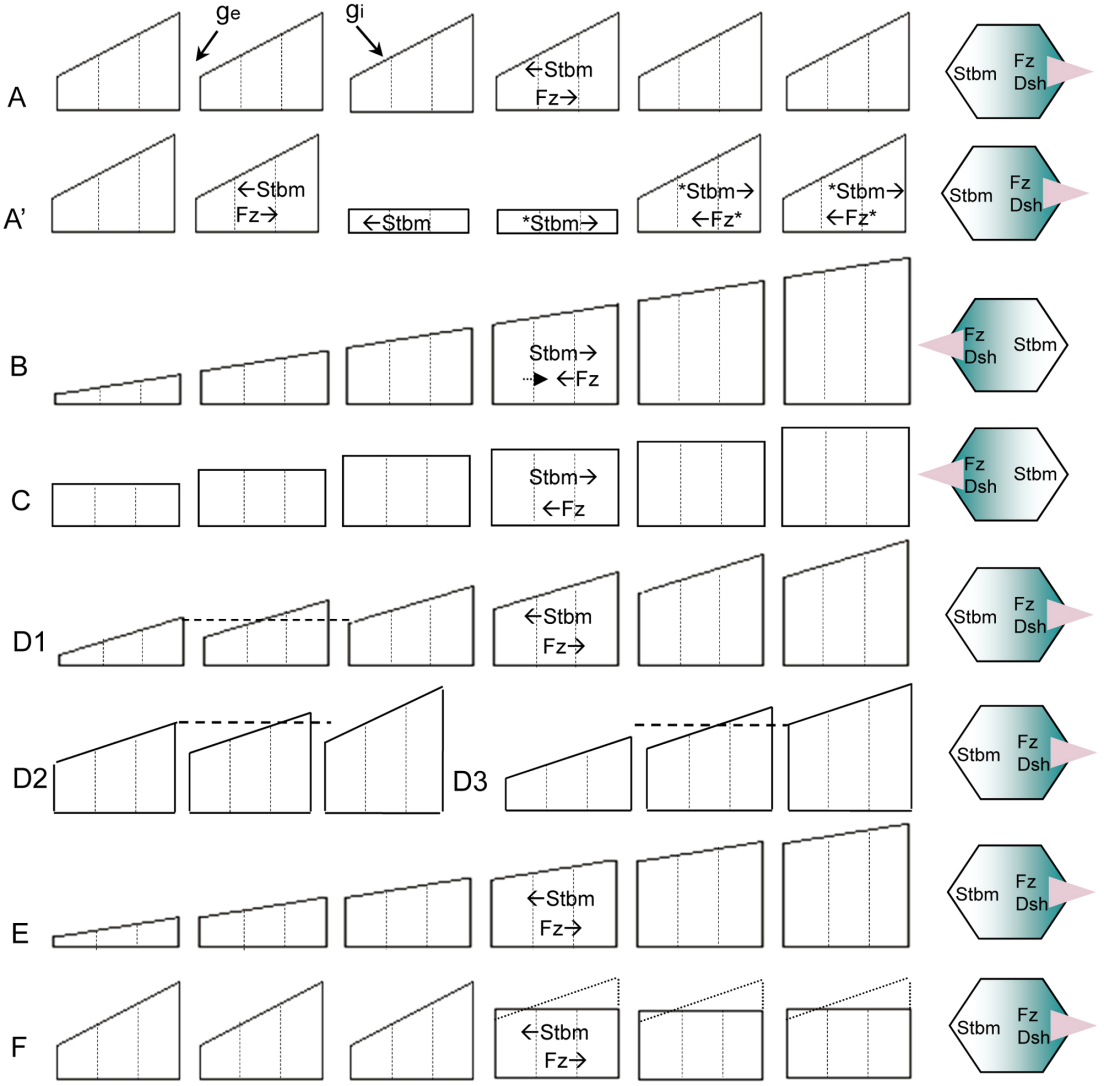
The six directional cues defined by g_i_ and g_e_, the Fz difference between intracellular and intercellular compartments. (A) g_i_ = 0.1 and g_e_ = −0.2 created an Fz gradient only within cells. (A′) Across the distal border of a clone of *fz* weak expression, reverse movement of Stbm and Fz (marked by *) occurred in wild-type cells. (B) g_i_ = g_e_ = 0.1 created an Fz gradient within and between cells. (C) g_i_ = 0.0 and g_e_ = 0.1 created an Fz gradient only between cells. (D1–D3) g_i_ = 0.1 and g_e_ = −0.1, g_i_ = 0.1 and −0.2 < g_e_ < −0.1, and g_i_ = 0.1 and −0.1 < *g_e_* < 0.0 created an Fz gradient within and between cells. (E) g_i_ = 0.1 and g_e_ = 0 created a global Fz gradient. (F) g_i_ = 0.1 and g_e_ = −0.2 in the three most proximal cells. Two dashed lines indicate three compartments in a cell. Solid and dashed arrows indicate stable and transient molecular movement. Icons at the right show the final cell polarity.

Intuitively, polarized distributions of molecules in a cell and their propagation into cells can be modelled by intra- and inter-cellular molecular interactions. To explore details, we first examined the behaviour of the model around a mutant clone in a tissue under a specific directional cue shown in Figure 2A. According to annotated molecular interactions, the initial distal distribution of Fz in each cell drove Stbm to move proximally toward the high eFz (external Fz) concentration and Dsh to move distally toward the high Fz concentration (abbreviated as high eFz and high Fz hereafter), respectively (Fig. 1). The generated Stbm and Dsh gradients between and within cells, then, drove Fz to move further distally. The two positive feedbacks formed by Fz/Stbm and Fz/Dsh interactions continuously drove the movement of Fz, Stbm, and Dsh in each cell until their distributions became fully polarized.

**Figure 3:**
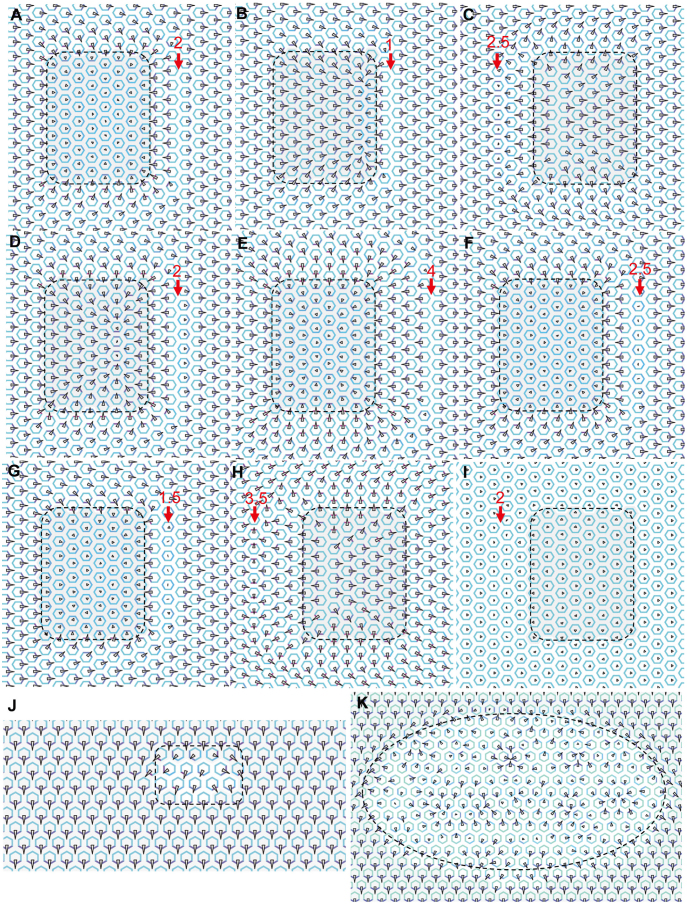
Different phenotypes of domineering non-autonomy around a mutant clone. Under the cue shown in Figure 2A, movement of Fz and Stbm was driven by the Fz and Stbm gradients within and between cells and was computed by Eqn 6D. The small arrow in each cell indicates the direction and length of hair (and the direction and degree of cell polarization). The red arrow in each picture indicates the boundary between the normally and reversely polarized cells, with the number indicating the row of reversely polarized cells. (A) Around a clone of *fz* weak expression. (B) Around a clone of slightly weak *fz* expression (87% of the normal concentration). (C) Around a clone of *fz* overexpression. (D) Around a clone of *stbm* overexpression. (E) Around a clone of *fz* weak expression in cells under a shallow cue (*g_i_* *= g_e_* = 0.01). (F) Around a clone of *fz* weak expression in cells with reduced mobility (ε = 0.001). (G) Around a clone of *fz* weak expression in a background of *stbm* weak expression. (H) Around a clone of *fz* overexpression in a background of *fz* weak expression. (I) Around a clone of *fz* overexpression in a background of *dsh* weak expression. (J) Cell polarization in and around a small clone was neatly aligned with nearby normal cells. (K) Cell polarization in and around a large clone was randomly organised.

Within a clone of *fz* weak expression (10% of the normal concentration), the sharp difference in the Fz concentration cross the clone boundary drove Stbm to move proximally at the proximal side, but distally at the distal side, toward the high eFz (Fig. 2A′). At the clone's distal outside, because the large eFz difference across the clone (driving Stbm in the first row wild-type cells to move distally) was against the eFz difference in wild-type cells (driving Stbm in the second and further rows wild-type cells to move proximally) (Fig. 2A′), the Stbm movement in the second row wild-type cells was reversed (Fig. 2A′). The reversed Stbm movement, via the coupled Fz/Stbm and Fz/Dsh interactions, not only drove Fz and Dsh to move against their initial direction but also penetrated into multiple rows of wild-type cells. At the clone's proximal outside, because the eFz difference across the clone was in the same direction as the eFz difference in wild-type cells, Stbm in wild-type cells moved proximally and no reversion of movement occurred (Fig. 2A′). As previously revealed^28^,^29^,^30^,^41^, a few rows of wild-type cells at the distal outside of the clone reversely polarized (Fig. 3A).

Outside a clone of *fz* overexpression (200% of the normal concentration), interactions between these molecules produced the opposite phenotype - domineering non-autonomy occurred in wild-type cells proximal to the clone with hairs pointing away from the clone (Fig. 3C)^18^,^41^. Further, as experimentally observed^11^,^18^, molecular interactions around a clone of *stbm* overexpression produced a phenotype analogous to that produced by a clone of *fz* weak expression (Fig. 3D). Finally, we examined the effect of clones in different genetic contexts. A clone of *fz* weak expression in a background of *stbm* weak expression caused hairs in some wild-type cells to point inward regardless of the weak *stbm* expression (Fig. 3G). A clone of *fz* overexpression in a background of *fz* weak expression produced a phenotype similar to that obtained with *fz* overexpression in a normal background (Fig. 3H). These results also agree with some experimental observations (the clone of *fz* weak expression in a background of *stbm* weak expression is comparable to a *fz*^−^ clone in a *pk*^−^ background in^30^), giving the model reasonable support. We also note that under *in vivo* conditions domineering non-autonomy shows certain variances. For example, different from simulation results, it was reported that a twofold increase in Fz had no observed effect on hair polarity in *Drosophila's* abdomen, which could be due to differences in molecular contexts (including the cues and the Ft/Ds/Fj system)^25^.

### PCP phenotypes were reproduced under multiple directional cues

PCP is observed in disparate tissues in diverse animals, but how interactions between the core components work with specific molecular contexts remains poorly explored. After examining four other cues shown in Figure 2BCDE, we found that the phenotypes acquired under the cue shown in Figure 2A were reproduced under these cues.

Under the cue shown in Figure 2B, the small initial intracellular and intercellular Fz gradients made both Dsh and Stbm move distally. The polarized Stbm distribution then outperformed the polarized Dsh distribution to cause Fz to move proximally against its initial intracellular gradient. Subsequently, the proximal movement of Fz reinforced the distal movement of Stbm, generating domineering non-autonomy at the proximal side of the clone of *fz* weak expression (Fig. S1A). Under the cue shown in Figure 2C, Stbm also moved distally, and the polarized Stbm distribution then caused the continuous proximal movement of Fz, resulting in domineering non-autonomy also at the proximal side of the clone of *fz* weak expression (Fig. S1B). Under the cue shown in Figure 2E where no intercellular Fz difference existed, the intracellular Fz gradient caused Fz and Dsh to move distally, which in turn caused Stbm to move proximally and domineering non-autonomy to occur at the clone's distal side (Fig. S1E).

We considered three potential cases of the cue shown in Figure 2D. In the first case a cell had equal eFz at the proximal and distal sides (Fig. 2D1), and initially only Dsh moved distally. The distal movement of Dsh then carried Fz to the distal side. In the second case a cell had higher eFz at its proximal side (Fig. 2D2), and from the beginning Stbm moved proximally and Dsh moved distally. In the third case a cell had higher eFz at its distal side (Fig. 2D3), and driven by the intracellular and intercellular Fz gradients, Dsh and Stbm both moved distally. As Fz accumulated in each cell's distal side, it later drove Stbm in adjacent cells to move proximally. So, all the three cases produced distally polarized cells and domineering non-autonomy at the clone's distal side (Fig. S1CD, Fig. S2).

The directional cues in Figure 2BCDE all have a linear proximal-to-distal gradient. Le Garrec *et al*. used an exponential distal-to-proximal gradient of the Fz ligand to generate an exponential distal-to-proximal gradient of Fz activity^29^. To examine whether directionality and nonlinearity of a cue affect PCP, we changed *g_i_* and *g_e_* (see Fig. 1 and Methods) to produce an exponential distal-to-proximal initial gradient of the Fz. Simulated phenotypes qualitatively equal to those produced under above linear cues. However, an unreported finding is that, if molecular movement was concentration-independent (computed with Eqn 6AB), the depth of the domineering non-autonomy became position dependent - it was deeper at the shallower region of the cue because cells in this region needed more time to become polarized, and this allowed reverse cell polarization to penetrate into more rows of wild-type cells.

We finally examined the cell-cell relay process that allows polarized distributions of molecules to propagate into cells^42^. Under the cue shown in Figure 2F cell polarization indeed propagated into all cells. But, outside an *fz* mutant clone, reverse cell polarization propagated into all wild-type cells distal to the clone, because the propagation was not resisted by normal polarization of cells in this region (Fig. S1F). Since domineering non-autonomy is always observed in a few rows of wild-type cells, a global cue should be required for PCP.

### Multiple factors influence the depth of domineering non-autonomy

Previous computational studies investigated the location and direction of domineering non-autonomy^28^,^29^, but largely overlooked quantitative differences in phenotypes. We specifically examined how the severity of gene mutation, the slope of directional cues, and the computation of molecular movement would influence the depth of domineering non-autonomy.

#### The severity of gene mutation

Around a clone of slightly weak *fz* expression (87% of the normal concentration), the mild Fz difference across the clone boundary drove slower reverse molecular movement in wild-type cells and generated fewer rows of domineering non-autonomy (Fig. 3B, compare with Fig. 3A).

#### The slope of the directional cue

A shallow cue would provide a weak driving force for the normal molecule movements, and accordingly, a weak resistance to the propagation of reverse molecule movement. In contrast, a sharp cue would do the opposite. We examined multiple shallow cues in different forms and, as expected, found that these cues all led to deeper domineering non-autonomy. Under a cue 10-times shallower than the cue shown in Figure 2A (Fz = 10.00/10.01/10.02 in a cell), domineering non-autonomy became much deeper (Fig. 3E, compared with Fig. 3A).

#### Speed of molecular movement

When cells had a 10-times smaller mobility (ε = 0.001) under the cue shown in Figure 2A, the domineering non-autonomy was slightly deepened (Fig. 3F, compared with Fig. 3A), because, as the speed of normal cell polarization became slower, it allowed the domineering non-autonomy to penetrate into more cells. If molecules move via microtubules^37^ and the capacity of microtubules is saturable, a small saturation threshold (λ = 0.02) would significantly deter PCP.

#### Molecule-specific mobility

In addition that all molecules responded equally to driving forces (ε = 0.01), we allowed each protein to have a specific mobility value. Under the cue shown in Figure 2A, when Stbm's response to eFz was reduced to 1/20 (ε = 0.0005), depth of domineering non-autonomy was reduced from 2 to 1 (Fig. S3A, compared with Fig. 3A), because Stbm's weakened response to Fz downplayed the role of the Fz gradient. When Fz's response to Dsh was reduced to 1/20 (ε = 0.0005), depth of domineering non-autonomy was slightly deepened from 2 to 3 (Fig. S3B, compared with Fig. 3A), because Fz's weakened response to Dsh slowed down the normal cell polarization. Under the cue shown in Figure 2B, when the response of Fz and Stbm to each other was doubled (ε = 0.02), domineering non-autonomy propagated into fewer rows of cells (from 4 in Fig. S1A to 3 in Fig. S3D), because the enhanced Fz/Stbm interaction significantly accelerated the normal cell polarization, which in turn deterred the propagation of domineering non-autonomy. The impact of mobility changes may be cue-dependent. Under the cue shown in Figure 2B, when Stbm's response to eFz was reduced to 1/20, not only the depth of domineering non-autonomy was reduced from 4 to 1, but also its location was shifted from the proximal side (Fig. S1A) to the distal side of the *fz* clone (Fig. S3C), because the substantially weakened response of Stbm to Fz failed to cause Fz to move proximally. These results should help explain quantitative differences in domineering non-autonomy.

### Intracellular signalling shows specific impact on cell polarization

If the intercellular Fz/Stbm interaction alone can ensure the propagation of cell polarization in wild-type cells^43^, what is the impact of intracellular molecule interactions on the propagation of cell polarization? With a clone of *fz* overexpression in a background of *dsh* weak expression (Dsh's role was reduced), simulations under different cues revealed that the polarization of cells near the clone was slowed down more significantly than the polarization of cells remote to the clone, and in some cases a slightly shallower domineering non-autonomy was observed (Fig. 3I, compared with Fig. 3C). This was because cells under strong driving forces (close to the clone boundary) were more significantly affected by impaired intracellular signalling than cells under weak driving forces (remote to the clone boundary), making reverse cell polarization propagate into fewer rows of cells. It was indeed found that polarity defects caused by a clone of *fz* overexpression in a *dsh* mutant background only propagate a short distance, and polarity defects in a *pk;dgo* double mutant background propagate over a shorter distance than in a *pk* mutant background^43^. Thus, the disproportionate impact of impaired intracellular signalling on cells near and distant to a clone enriches PCP phenotypes.

To examine the role of a second intracellular feedback, the Stbm/Pk interaction was added with the same parameters as the Fz/Dsh interaction, which promotes the proximal movement of Stbm and Pk in cells^13^,^18^. Under varied conditions the Stbm/Pk-conducted feedback only slightly reduced the time period of PCP (Table S3). When Stbm and Pk had a larger ε, Pk's role was enhanced, but not in a linear way (Table S3). When the Stbm/Pk interaction was treated as the sole intracellular feedback, it did not speed up PCP as effective as the Fz/Dsh interaction, because Dsh interacts with the leading component Fz (Table S3). These results suggest that the second and third intracellular feedbacks may inherently play a less important role, which agrees with the experimentally identified order of importance Dsh > Pk > Dgo (Dgo is the third intracellular component)^43^.

### Propagation of cell polarization corrects local errors in cues

In *Drosophila* wing PCP signalling can align cells' polarization to overcome local errors in the directional cue^21^, but in large clones errors can cause long-range and irregular propagation of PCP signalling^44^. We examined to what extent error correction is a general property.

**Figure 4:**
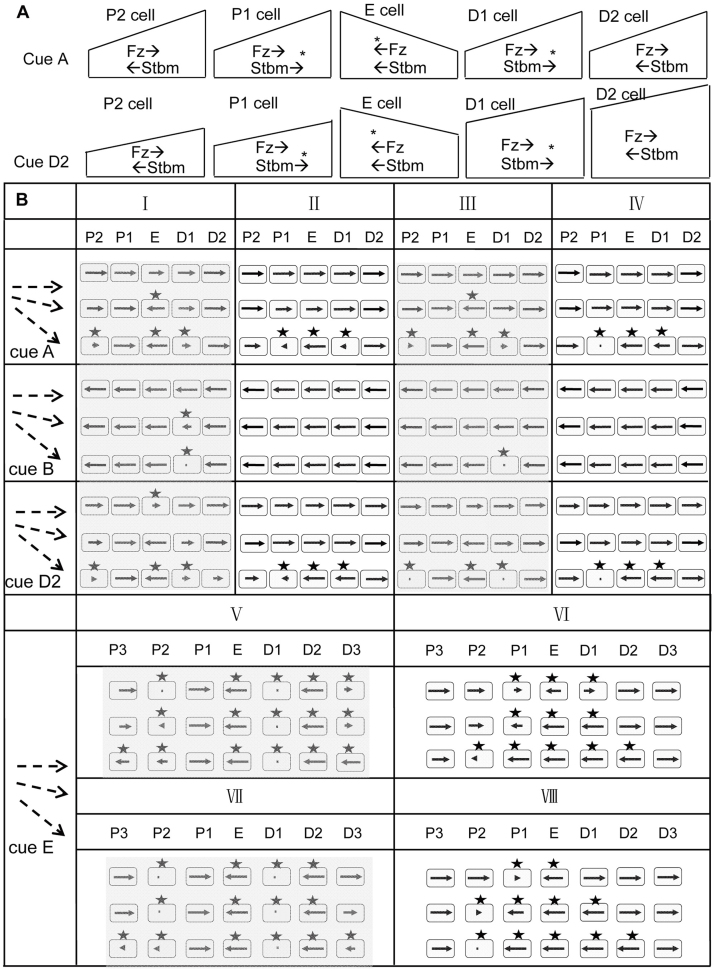
Error correction under different situations. (A) For the 5 cells in a row, E cell, P2 cell, P1 cell, D1 cell and D2 cell indicate the error cell, its second and first proximal neighbours, and its first and second distal neighbours. In the E cell, Fz had a reversed gradient under the cues shown in Figure 2A (top panel) and in Figure 2D2 (bottom panel). The reversed Fz gradient in the E cell caused wrong Stbm movement (marked by *) in neighbouring cells, which, via coupled intra- and intercellular signalling, either propagated into more neighbouring cells or was gradually corrected. (B) Error correction occurred (in 5 or 7 cells in a row) under all conditions examined. The title column shows the flat, reversed and sharply reversed Fz gradient in the E cell. In the heading rows, P2/P1/E/D1/D2 indicate the error cell and its proximal and distal neighbours, I/II/V/VI indicate results computed using Eqn 6A, and III/IV/VII/VIII indicate results computed using Eqn 6C. Shadowed and un-shadowed results were computed with uncoupled and coupled intracellular movement of Fz and Stbm, respectively. In each panel a cell is represented by a round square. In a cell a small arrow indicates the direction and degree of cell polarization, but a dot or a triangle indicates the cell is unpolarized or poorly polarized, and an asterisk indicates uncorrected errors. The figure shows that if the error in the E cell was a flat Fz gradient, in most cases it was corrected; but if the error was a sharply reversed Fz gradient, in many cases it propagated into neighbouring cells. In all cases the coupled intracellular movement of Fz and Stbm significantly improved the performance of error correction (the un-shadowed parts).

The local error, in a column of error cells (E cell), was assumed to be a flat or reversed Fz distribution (Fig. 4). Under the cue shown in Figure 2A, according to the outlined molecular interactions, to correct the wrong proximal movement of Fz and Dsh in the E cell, persistent proximal movement of Stbm in the D1 cell was required to drive Fz and Dsh move distally in the E cell. A mild error made the E cell polarize slower than its distal neighbours, allowing the Fz gradient across the D1 cell to drive persistent proximal movement of Stbm in the D1 cell, and consequently, to correct the wrong Stbm movement in the E cell. If the error was severe, the E cell was polarized faster than its distal neighbours and the wrong Fz and Dsh movement propagated into these distal neighbours instead of being corrected (Fig. 4B). Under the cue shown in Figure 2B, the reversed Fz distribution in the E cell agreed with the final cell polarity but caused wrong molecule movement in its distal neighbours. Likewise, if the error was severe, the wrong polarization in the D1 cell could not be corrected (Fig. 4B). We found that under all the cues, regardless of the methods of computing molecule movement, a severe local error propagated into a few rows of normally polarizing wild-type cell.

We next examined the situation around an *fz* mutant clone, within which the initial Fz concentration was assumed to be a random value between 10.0 and 10.1 in each cell and outside which cells were under the cue shown in Figure 2A. With a large clone (19 × 24 cells), cells in the clone were not aligned, but propagation of cell polarization resulted in locally organised cell polarity (Fig. 3K) (typical swirling patterns did not occur because of the normal cell geometry). With a small clone (4 × 4 cells), polarization of cells in the clone was aligned by nearby wild-type cells (Fig. 3J). As error correction penetrating into cells in the clone, slower signalling was found to result in poorer local organisation and more irregular PCP phenotypes, which is a property irrespective of clone size.

### Coupled intracellular Fz and Stbm movement can significantly enhance error correction

The recognized Fz and Stbm interaction and movement (Fz and Stbm move toward high eStbm and high eFz in each cell^18^,^30^) do not preclude that Fz and Stbm may move toward the same direction. In simulating error correction, if Fz and Stbm movements were determined independently by eStbm and eFz, in some situations Fz and Stbm moved in the same direction in some cells, which significantly impeded error correction. We performed further simulations with the extra condition that Fz and Stbm movement was also driven by the low Stbm and low Fz within a cell (see Eqn 2C, Eqn 3C, Fig. 1D). Under the four cues shown in Figure 2ABD2E, this condition significantly enhanced the power of error correction (Fig. 4B). Even if in some cases the error was not completely corrected, its propagation was strongly restrained (compare column V with VI and column VII with VIII in Fig. 4B). More importantly, this condition reduced the amount of time required for error correction, making simulation results fit the finding that error correction does not require additional time^21^.

## Discussion

PCP signalling produces diverse phenotypes in different tissues in animals from insects to vertebrates, but how these phenotypes are generated at the cell level remains controversial (reviewed in^1^,^5^,^6^). Computational models have been used to help explore mechanisms of PCP signalling and patterning. We used this model to examine how intercellular and intracellular feedbacks behave under different molecular contexts, which have not been adequately examined so far, and revealed how details of PCP phenotypes, especially the depth of domineering non-autonomy, are determined by details of feedback mechanisms and directional cues. Although all phenotypes generated upon known molecular interactions have been observed in experiments, some results are insightful instead of being merely hindsight, providing sensible explanations for some previously observed subtle aspects of PCP. For example, domineering non-autonomy propagated longer under a weak directional cue, because such a cue would present a weak resistance to the propagation of reverse cell polarization, which may explain the finding that loss of *ft* and *ds* (which would weaken the cue) over a large region deepens *fz* domineering non-autonomy^20^,^21^,^25^. Around an *fz* mutant clone in a background of *dsh* weak expression, domineering non-autonomy propagated over a shorter distance, because impaired intracellular signalling more significantly affects cells (undergoing reverse polarization) near the clone. Indeed, it was observed that around a clone of *fz* overexpression in wings mutant for *dsh* cell polarity defects only propagated at most 3–4 cells^43^. If there were defects in a directional cue, similarly, slower normal cell polarization would allow irregular PCP to propagate longer. The comparative levels of Fz in cells were suggested to mainly determine PCP propagation^30^, and an experimental study identified the order of importance Dsh > Pk > Dgo^43^. Data produced by our simulations suggest that the second and third intracellular feedbacks may inherently play a less important role than the first, Dsh-participated feedback (Table S3). Further, results of simulating error correction raise the prediction that the movement of Fz and Stbm may also be determined by Fz and Stbm concentrations within each cell.

Reversed cell polarity around a clone of *fz* weak expression occurs at the distal side in *Drosophila* wings and at the posterior side in *Drosophila* abdomen compartments^30^. Simulations showed that under some cues (shown in Figure 2BC), reversed cell polarity around the *fz* clone occurred at the opposite (proximal) (Fig. S1). We do not suggest that this may occur in *Drosophila* wing or abdomen. Given that in all situations reversed cell polarity occurs at the side hairs normally point to (Fig. 2), we think that different sides do not indicate inconsistency, and that in certain tissues of some animals hair directions may point to, and reversed cell polarity occurs, at the side of low initial Fz gradient. Since in both *Drosophila* wing and abdomen reversed cell polarity around a clone of *fz* weak expression occurs at the side with high Wingless concentration, more generally the gradient of Wingless, together with Ft, Ds, and Fj, instead of Fz itself, may determine the side of domineering non-autonomy.

**Figure 5:**
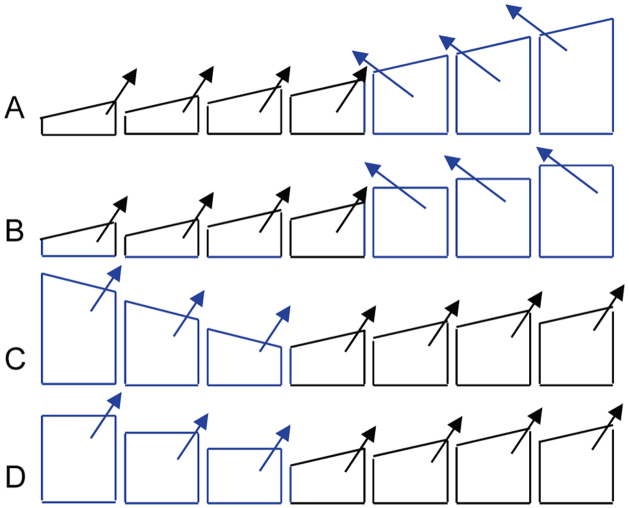
Increased *fz* expression may have different impact on hair directions at the distal and proximal sides in the wing under the cue shown in Figure 2D. The arrow in each cell indicates the final cell polarity. When the Fz gradient is substantially strengthened at the distal side (illustrated by the three distal cells in blue) it would produce a gradient shown in (A) or (B), which would cause cells at the distal side to point proximally. When the Fz gradient is substantially strengthened at the proximal side (illustrated by the three proximal cells in blue) it would produce a gradient shown in (C) or (D), which would maintain cells at the proximal side to point distally.

If a simplified computational model embodies the inherent properties of a signalling system, simulations would make these properties be uncovered through reproduced phenotypes under varied conditions^40^. The simulations of multiple phenotypes of domineering non-autonomy under varied conditions suggest that both the propagation of wrong cell polarization and the propagation of error correction are a damped process that is gradually stopped by the correct and wrong polarization of cells (they should also be stopped, or much impeded, by compartment boundaries in the wing and abdomen, see Fig. 5 in^45^). Damped propagation not only explains varied depth of domineering non-autonomy but also associates error correction with domineering non-autonomy. It highlights that behind diverse phenotypes the basic mechanisms of patterning can be quite simple. More to do will be to examine whether the propagation of signalling produced by the Ft/Ds/Fj system would have the same feature^46^ (a recent work indeed indicates that Fat2 is involved in the propagation of a global cue in the *Drosophila* ovary^34^).

The cue shown in Figure 2A concords with that no initial tissue-scale Fz gradient is found in the wing^47^, but those shown in Figure 2BCDE seemingly not. By setting a specific parameter, Amonlirdviman *et al* enabled a flat Fz distribution to generate biased Fz activity in each cell to push the reactions of Fz binding toward the desired direction^28^. By assuming an exponential gradient of an Fz ligand instead of Fz *per se*, Le Garrec *et al* realized controlled Fz signalling in cells^29^. However, it is difficult for a flat Fz distribution or a gradient of Fz ligand to explain the observation that the artificially increased *fz* expression at the wing's distal region reverses the polarity of up to 50 rows of cells at the proximal border of this region, but the artificially increased *fz* expression at the wing's proximal region does not change cell polarity (Fig. 5 in^41^). Since a clone of *fz* overexpression usually causes polarity reversal in just a few rows of cells (Fig. S8E in^28^ and Fig. 2BC in^48^), the polarity reversal in up to 50 rows of cells should be caused not only by the artificially increased *fz* expression at the wing's distal region, but also by the gradient of Fz at the proximal border of the distal region^41^. Can an initial global Fz gradient, for example, shown in Figure 2D, explain the polarity reversal in 50 or so rows of cells? We postulate that, if the artificial increase of *fz* expression significantly increases the Fz concentration at the distal region, the Fz gradient at the region's proximal side (abutting the unchanged proximal region) will unlikely remain the form of Figure 2D, but more likely resembles the form of Figure 2B or Figure 2C. In either case, as simulations indicated, hairs at the region's proximal side would have a reversed direction (blue cells in Fig. 5AB). On the other hand, if the artificial increase of *fz* expression significantly increases the Fz concentration at the proximal region, the Fz gradient at the region's distal side will produce cell polarity shown in Figure 5C or Figure 5D, that is, cells maintain their distal hair direction.

Upon simulation results we propose that the initial Fz gradient in the wing is in the form of Figure 2D. If the cue shown in Figure 2D exists in *Drosophila* wing, it challenges the finding that a global Fz gradient was not found in the wing^47^. A possible solution to the contradiction is that the initial Fz gradient may be quite shallow. A shallow intercellular gradient of Fz or Fz activity has been widely assumed^49^,^50^, yet how shallow it could be remains unclear. Small differences, as little as 2%, between cells can make receptors activated and cells polarized^51^, and our simulations showed that shallow Fz gradients in the forms shown in Figure 2BCD (from 10.03 to 11.16 at the proximal and distal ends of the cell space) effective produced normal and mutant phenotypes. Such shallow gradients may not be detected or detectable in experiments. Recently it was found that the Fz extracellular domain is a ligand for Stbm during non-autonomous planar cell polarity signaling^48^, this lends further support for the possibility of a shallow Fz gradient. According to simulations, a direct consequence of shallow Fz gradients may be rich and large-scale patterns of abnormal hair directions, as observed not only in *Drosophila* but also in vertebrates^52^. Moreover, if the Fz gradient shown in Figure 2D forms first at the distal side and then stretches proximally to form a distal-to-proximal gradient in the wing, it may lend an explanation for the distal-to-proximal asynchronous pre-hair differentiation (Fig. 3 in^45^).

## Methods

### Cell array

A lattice of 114 × 114 computational units was defined, each representing a cell compartment. Six compartments defined a hexagonal epithelial cell and shared a unique ID (Fig. 1AB). A mutant clone contained 8 × 9 cells. Weak and over expression of a gene was represented by reduced and increased protein concentrations (10% and 200% of the normal value, respectively).

### Molecular concentrations

Similar to previous models in which dimensionless values were used as molecular concentrations^28^,^29^, we arbitrarily chose 10.0 as the concentration of Fz, Dsh, Stbm, and Pk. Simulations confirmed that the model tolerated a great range of concentration settings (Table S2).

### Directional cues

The global PCP pattern in the wing is gradually reoriented toward the distal side^38^. But, (as in previous models) we assumed that in the simulated time period the cue and cell polarization are in the same directions. Different values of *g_i_* and *g_e_*, two parameters defining the differences in Fz concentration between two proximodistally connected intracellular and intercellular compartments, defined five initial Fz distributions to act as the directional cue (some could be biologically unrealistic) (Fig. 2). In the whole lattice the Fz concentration ranged from 10.3 (in the most proximal cell) to 21.6 (in the most distal cell) in Figure 2BCD and from 10.3 to 17.8 in Figure 2E.

### Molecular movement

U_[*x,y*]_ was U's concentration in the compartment [x,y]; eU_[*x,y*]_ was U's concentration in [x,y]'s abutting foreign compartment; In_[x,y,t]_ was the amount of U moved into [x,y] at time step *t*; and Out_[x,y,t]_ was the amount of U moved out of [x,y] at time step *t* (Fig. 1C). The concentration of U at [x,y] at time t + 1 was 
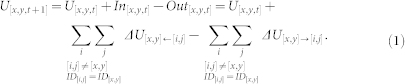
As 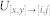 was computed in every compartment, 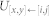 was computed simultaneously. The gradients of molecules (*driving forces*) that interact with U and U's mobility in response to these gradients (*mobility*) determined the direction and amount of U movement. In detail, Fz moved from [x,y] to [i,j] driven by the Stbm gradient in the two external compartments abutting [x,y] and [i,j] (the black and green eStbm in Fig. 1C), and by the Dsh and Stbm gradients in the compartment [x,y] and [i,j] (the black and red Dsh and Stbm in Fig. 1C). The driving forces for 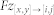 were 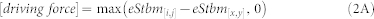

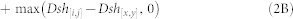

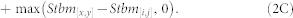
(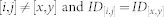). They drove Fz to move toward the *high* eStbm, the *high* Dsh, and the *low* Stbm (the big eStbm, big Dsh, and small Stbm in Fig. 1D). Similarly, Stbm moved from [x,y] to [i,j] driven by the Fz gradient in the two external compartments abutting [x,y] and [i,j], and by the Fz and Pk gradients in the compartment [x,y] and [i,j]. The driving forces for 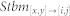 were 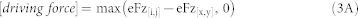

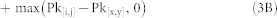

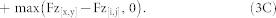
They drove Stbm to move toward the *high* eFz, the *low* Fz, and the *high* Pk. Because Dsh movement was driven by the Fz gradient in the compartment [x,y] and [i,j] toward the *high* Fz, the driving force for 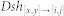 was 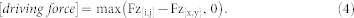
And because Pk movement was driven by the Stbm gradient in the compartment [x,y] and [i,j] toward the *high* Stbm, the driving force for 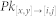 was 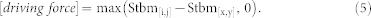
In these equations *max*() reported the larger value of its two parameters. By Eqn 2B and Eqn 4, Fz and Dsh form the first intracellular feedback; and by Eqn 3B and Eqn 5, Pk and Stbm form the second intracellular feedback. Eqn 2C and Eqn 3C make Fz and Stbm form the third intracellular feedback and make Fz and Stbm movement directly driven by intracellular Fz and Stbm gradients.

We explored different definitions of *mobility*, including all molecules had a fixed mobility value 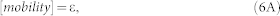
each molecule U had a specific, concentration-independent mobility value 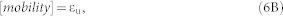
each molecule U had a concentration-dependent, unsaturable mobility value 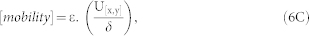
and each molecule U had a concentration-dependent, saturable mobility value 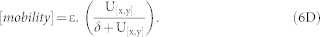
We also examined 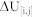 limited by a threshold λ 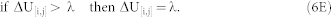
In above equations and in simulations, by default ε = 0.01, δ = 10.0, and Eqn 6D was adopted. Except a very small λ in Eqn 6E, different ε and δ did not produce qualitatively different results (Table S1).

### Hair direction

The direction and length of the hair in each cell were determined by the vector sum of the Dsh distribution in six compartments. Hair length indicated the degree of cell polarization. PCP phenotypes were represented by tissue-scope hair directions.

### Stability of phenotypes

PCP phenotypes were captured when in 96% of the cells Fz reached a typical proximodistal distribution (Fz concentration < 0.5 in the proximal and central compartments, Fig. 1 and Table S1). This proximodistal distribution is stable in that reverse molecule movement is prohibited by positive feedbacks, but unstable in that the trivial Fz (and Dsh, Stbm) concentration difference between the two distal compartments would gradually drive Fz to move finally into one distal compartment (Fig. 1B; Table S1). This is caused by that we artificially divide the distal (and proximal) side into two independent computational units. Under *in vivo* condition, if molecular movement subjects to the concentration-dependent saturating kinetics, the typical proximodistal distributions of molecules become more stable (Table S1).

### Implementation

The model was implemented under Linux by MATLAB.

## Author Contributions

H.Z. and M.O. designed research; H.Z. performed simulations; H.Z. and M.O. analyzed data and wrote the paper.

## Supplementary Material

Supplementary Information Supplementary Information
