# Supplementary material for: Damped propagation of cell polarization explains distinct PCP phenotypes of epithelial patterning
Source: Sci Rep. 2013 Sep 2;3:2528. doi: 10.1038/srep02528 (PMC3759060; doi:10.1038/srep02528)
Supplement: Supplementary Information [file srep02528-s1.pdf]

Damped propagation of cell polarization explains distinct PCP phenotypes of epithelial patterning

Author: Hao Zhu *
Bioinformatics Section, School of Basic Medical Sciences,
Southern Medical University, Guangzhou, 510515, China
Email: hao.zhu@ymail.com
Phone: +86 (0) 20 616 48632
Fax: +86 (0) 20 616 48299

Author: Markus R. Owen *
Center for Mathematical Biology and Medicine, School of Mathematical Sciences
The University of Nottingham, Nottingham, NG7 2RD, UK
Email: markus.owen@nottingham.ac.uk
Phone: +44 (0) 115 84 67214
Fax: +44 (0) 115 95 13837

Table S1. Binding between molecules stabilises the Fz/Dsh/Stbm/Pk complex and cell polarity

		Time1(T1)	Time2(T2)	Time3(T3)	Time4(T4)	Time5(T5)	Time6(T6)	Time7(T7)
1	$(\varepsilon\cdot G)$	= 74 steps	= 95 steps	= T2*1.5	= T2*2.0	= T2*2.5		
		.5/10.2/19.0<br>.5/10.2/19.0	.5/.5/29.8<br>.5/.5/29.6	.5/.5/31.3<br>.5/.5/29.4	.5/.5/34.0<br>.5/.5/26.8	.5/.5/43.2<br>.5/.5/17.3		
2	$(\varepsilon\cdot G)\cdot\left(1-\frac{U^3}{20.8^3+U^3}\right)$	= 79 steps	= 104 steps	= T2*1.5	= T2*2.0	= T2*2.5	= T2*3.0	= T2*4.0
		.5/10.7/19.4<br>.5/10.7/19.4	.5/.5/30.5<br>.5/.5/30.3	.5/.5/30.7<br>.5/.5/30.2	.5/.5/30.9<br>.5/.5/30.0	.5/.5/31.2<br>.5/.5/29.8	.5/.5/31.6<br>.5/.5/29.4	.5/.5/32.9<br>.5/.5/28.0
3	$(\varepsilon\cdot G)\cdot\left(\frac{U}{\delta}\right)$	= 103 steps	= 122	= T2*1.5				
		.5/2.6/26.7<br>.5/2.6/27.2	.5/.5/28.7<br>.5/.5/30.6	.5/.5/5.0<br>.5/.5/55.1				
4	$(\varepsilon\cdot G)\cdot\left(\frac{V}{\delta}\right)\cdot\left(1-\frac{U^3}{20.8^3+U^3}\right)$	= 108 steps	= 129	= T2*1.5	= T2*2.0	= T2*2.5		
		.5/2.8/26.6<br>.5/2.8/26.8	.5/.5/29.5<br>.5/.5/29.9	.5/.5/29.4<br>.5/.5/31.1	.5/.5/26.8<br>.5/.5/33.7	.5/.5/11.1<br>.5/.5/49.0		
5	$(\varepsilon\cdot G)\cdot\left(\frac{U}{\delta+U}\right)$	= 166 steps	= 202	= T2*1.5	= T2*2.0			
		.5/5.2/24.3<br>.5/5.2/24.4	.5/.5/29.6<br>.5/.5/29.9	.5/.5/29.3<br>.5/.5/31.2	.5/.5/22.9<br>.5/.5/37.5			
6	$(\varepsilon\cdot G)\cdot\left(\frac{U}{\delta+U}\right)\cdot\left(1-\frac{U^3}{20.8^3+U^3}\right)$	= 176 steps	= 217	= T2*1.5	= T2*2.0	= T2*2.5	= T2*3.0	= T2*4.0
		.5/6.1/23.4<br>.5/6.1/23.5	.5/.5/29.7<br>.5/.5/29.8	.5/.5/30.1<br>.5/.5/30.4	.5/.5/30.1<br>.5/.5/30.3	.5/.5/29.9<br>.5/.5/30.7	.5/.5/29.6<br>.5/.5/30.9	.5/.5/28.1<br>.5/.5/32.5

The results were obtained under the cue shown in Figure 2A. To a molecule U, G is its driving force, U is its concentration, $\varepsilon = 0.01$ in all cases, and $\delta = 10.0$ in case 3 to case 6. At T2 (time point 2) over 96% of the molecules locates to the two distal or two proximal compartments. Numbers such as .5/10.7/19.4 indicate that Fz concentrations in the proximal, central and distal compartments are lower than 0.5, 10.7 and 19.4. Upon the recognition that molecular movement under *in vivo* conditions was often subject to the concentration-dependent saturating kinetics, simulations were performed where ε was multiplied by $1-\frac{K_{max}U^n}{(k^n+U^n)}$. Because Fz and Dsh bind to each other at the distal side within a cell and Fz binds to Stbm at the distal side between two cells, there should at least be n = 3. In addition, we made k = 20.8 and $K_{max} = 1$ so that the saturation effect was trivial in unpolarised and slightly polarised cells but strong when cells became nearly fully polarised. The new ε made polarised molecular distributions highly stable under varied conditions and significantly postponed the further movement of molecules in distal (proximal) components (compare the time when numbers with underlines occur in row 1, 3, 5 and in row 2, 4, 6).

Table S2. Valid ranges of initial molecular concentrations under different cues

	Fz	Dsh	Stbm
Cue shown in Fig. 2A	Any	Any	Any
Cue shown in Fig. 2B	Any	<15.0	>7.0
Cue shown in Fig. 2C	Any	Any	Any
Cue shown in Fig. 2D	Any	Any	Any
Cue shown in Fig. 2E	Any	>9.0	<11.0

Table S3. Impact of intracellular signalling on cell polarisation

Model components	T2	Time to reach T2	Methods
Fz/Stbm	83	100%	$[mobility] = \varepsilon$
Fz/Stbm/Dsh	66	79%	
Fz/Stbm/Dsh/Pk	65	78%	
Fz/Stbm/Dsh/Pk ($\varepsilon_{Pk} = \varepsilon_{Stbm} = 0.1$)	46	55%	
Fz/Stbm/Pk	79	95%	
Fz/Stbm	112	100%	$[mobility] = \varepsilon \cdot \left(\frac{U_{[x,y]}}{\delta} \right)$
Fz/Stbm/Dsh	81	72%	
Fz/Stbm/Dsh/Pk	80	71%	
Fz/Stbm/Dsh/Pk ($\varepsilon_{Pk} = \varepsilon_{Stbm} = 0.1$)	63	56%	
Fz/Stbm/Pk	109	97%	
Fz/Stbm	183	100%	$[mobility] = \varepsilon \cdot \left(\frac{U_{[x,y]}}{\delta + U_{[x,y]}} \right)$
Fz/Stbm/Dsh	136	74%	
Fz/Stbm/Dsh/Pk	133	73%	
Fz/Stbm/Dsh/Pk ($\varepsilon_{Pk} = \varepsilon_{Stbm} = 0.1$)	99	54%	
Fz/Stbm/Pk	177	97%	

The results were obtained under the cue shown in Figure 2D. $\varepsilon = 0.01$ and $\delta = 10.0$. In each setting of *mobility*, the time period for the basic model (consisting of only Fz and Stbm without intracellular signalling) to reach T2 (see Table S1) was 100. In all cases, the addition of intracellular signalling reduced the time for the cell to reach T2, but the second component Pk only slightly increased the speed of cell polarisation. If Stbm and Pk had a large ε, the impact is very significant. If Pk interaction was treated as the sole intracellular component, its contribution was not as significant as Dsh.

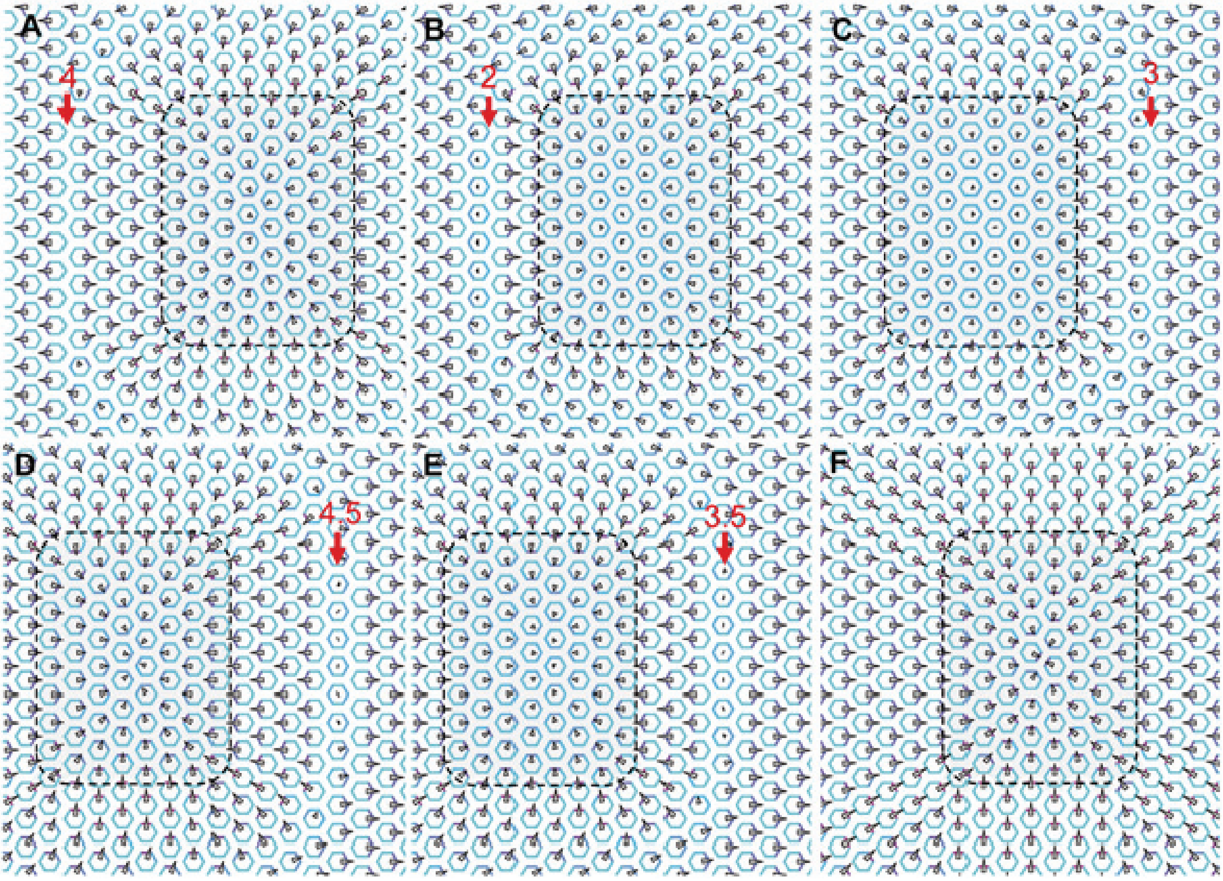

Figure S1 Different phenotypes of domineering non-autonomy around a mutant clone under different directional cues. The small arrow in each cell indicates the direction and length of hair (and the direction and degree of cell polarization). The red arrow in each picture indicates the boundary between the normally and reversely polarised cells, with the number indicating the layer of reversely polarised cells. (AB) Reversed hair directions at the proximal side of a clone of *fz* weak expression under the directional cue shown in Figure 2B and Figure 2C, respectively. (CDE) Reversed hair directions at the distal side of a clone of *fz* weak expression under the directional cue shown in Figure 2D1, Figure 2D2 and Figure 2E, respectively. (F) Reversed hair directions at the distal side of a clone of *fz* weak expression propagated into all cells at this side (the directional cue was shown in Figure 2F).

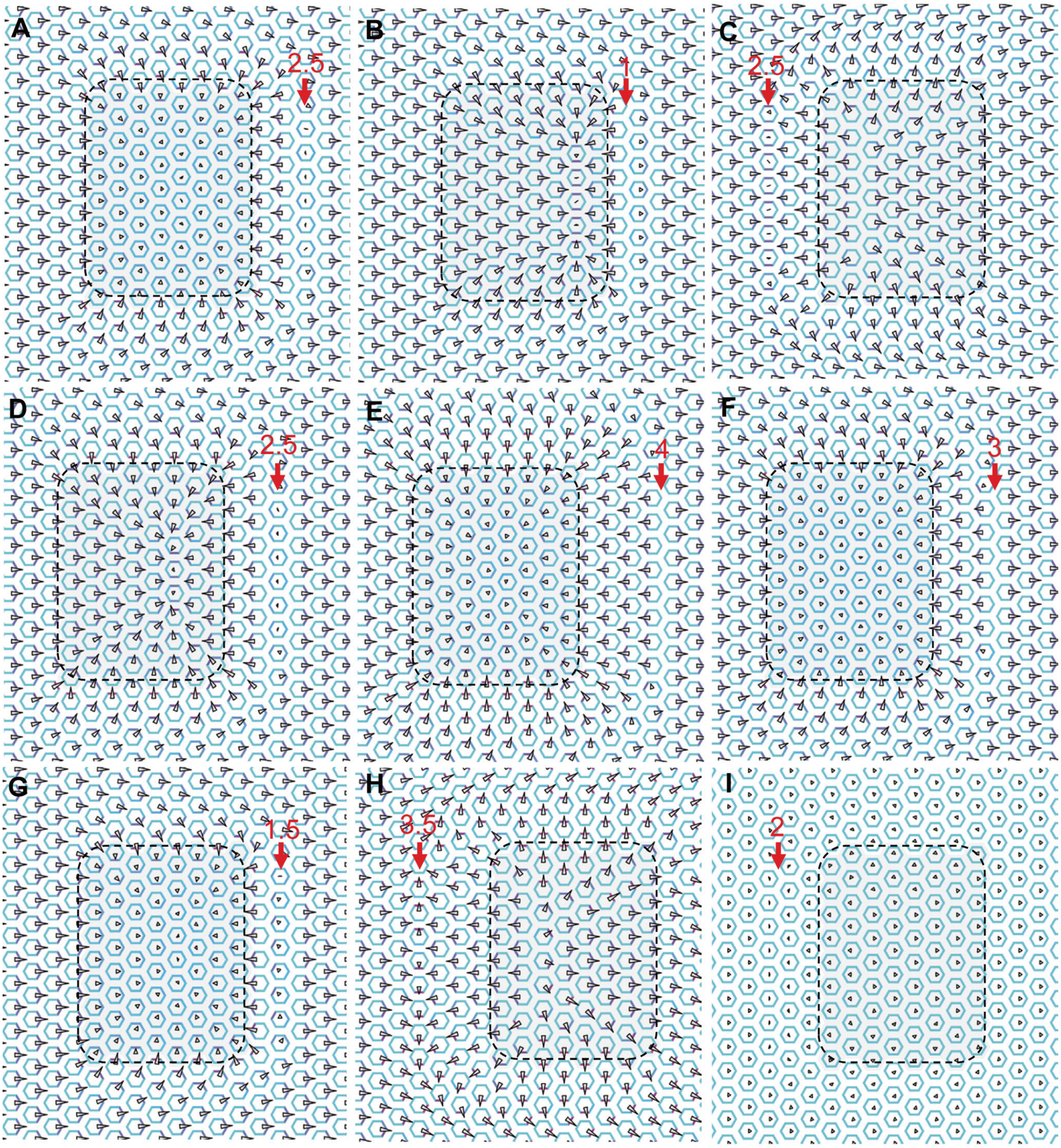

Figure S2 Different phenotypes of domineering non-autonomy around a mutant clone under the directional cue shown in Figure 2D2 (for a comparison with Figure 3). (A) Around a clone of *fz* weak expression. (B) Around a clone of slight weak *fz* expression (87% of the normal concentration). (C) Around a clone of *fz* overexpression. (D) Around a clone of *stbm* overexpression. (E) Around a clone of *fz* weak expression in cells under a shallow cue (10% of the normal cue concentration). (F) Around a clone of *fz* weak expression in cells with reduced mobility (ε = 0.001). (G) Around a clone of *fz* weak expression in the background of *stbm* weak expression. (H) Around a clone of *fz* overexpression in the background of *fz* weak expression. (I) Around a clone of *fz* overexpression in the background of *dsh* weak expression. Movement of Fz and Stbm was driven also by intracellular distributions of Fz and Stbm.

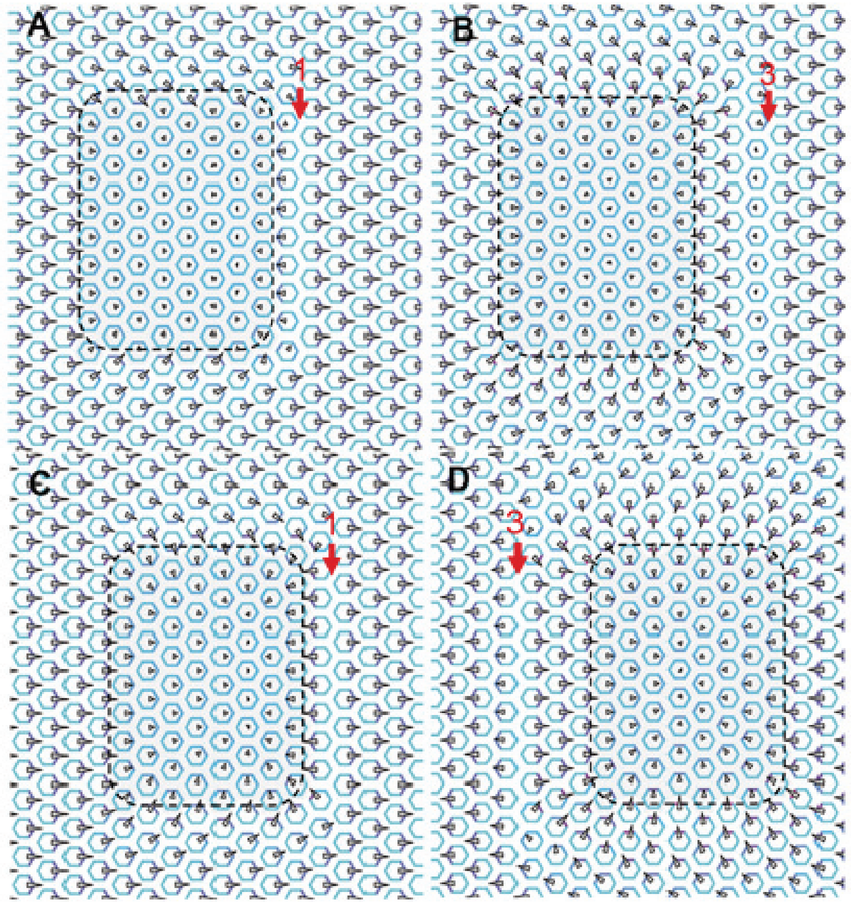

Figure S3 The impact of protein-specific mobility on domineering non-autonomy. (A) Stbm's response to the external Fz was reduced to 1/20 (ε= 0.0005) under the cue shown in Figure 2A. Domineering non-autonomy occurred at the right side. (B) Fz's response to Dsh was reduced to 1/20 (ε= 0.0005) under the cue shown in Figure 2A. (C) Stbm's response to external Fz was reduced to 1/20 (ε= 0.0005) under the cue shown in Figure 2B. (D) Fz and Stbm's responses to each other was doubled (ε=0.02) under cue shown in Figure 2B. Domineering non-autonomy occurred at the distal side of the *fz* clone in (A-C), but at the proximal side of the *fz* clone in (D).
